# M1 macrophages as promising agents for cell therapy of endometriosis

**DOI:** 10.1016/j.heliyon.2024.e36340

**Published:** 2024-08-14

**Authors:** Daria Artemova, Polina Vishnyakova, Andrey Elchaninov, Elena Gantsova, Gennady Sukhikh, Timur Fatkhudinov

**Affiliations:** a Avtsyn Research Institute of Human Morphology of Federal State Budgetary Scientiﬁc Institution “Petrovsky National Research Centre of Surgery”, 3 Tsurupa Street, 117418, Moscow, Russian Federation; b Research Institute of Molecular and Cellular Medicine, RUDN University, 6 Miklukho-Maklaya Street, 117198, Moscow, Russian Federation; c National Medical Research Center for Obstetrics, Gynecology and Perinatology Named After Academician V.I. Kulakov of Ministry of Healthcare of Russian Federation, 4 Oparina Street, 117997, Moscow, Russian Federation

**Keywords:** Endometriosis, Mouse model, Macrophages, Cell therapy, Polarization

## Abstract

Endometriosis is a chronic estrogen-dependent disease characterized by the presence of endometrial glands and stroma outside their normal anatomical location. While laparoscopic removal of foci remains the gold standard therapy, it has limited efficacy and certain risks. However, cell therapy using pro-inflammatory M1 macrophages presents a promising and minimally invasive alternative for treating endometriosis. This approach showcases the potential for innovative and effective treatments for this condition. This study aims to explore the anti-endometriosis properties of M1 macrophages. A reproducible syngeneic mouse model of endometriosis was utilized, revealing that formed foci are primarily composed of macrophages with an anti-inflammatory M2 phenotype rather than M1 macrophages. To investigate further, chemically reprogrammed M1 macrophages were labeled with the membrane fluorescent tag PKH26 and administered to animals with endometriosis. Therapy resulted in a decrease in the number and size of foci, accompanied by a shift in the phenotypic composition of peritoneal macrophages. Specifically, the content of M2 macrophages decreased while that of M1 macrophages increased, resembling the composition of healthy animals. Our study conclusively demonstrates the anti-endometriosis properties of M1 macrophages, providing a strong foundation for future research in the cell therapy of endometriosis.

## Highlights

• Сell therapy is a promising treatment option for endometriosis.
• Administration of M1 macrophages causes regression of endometriosis foci.
• M1 macrophages administration causes a shift in the balance to M1 macrophages.
• After therapy, the peritoneal macrophages phenotype corresponded to healthy mice.
• The shown M1 macrophages properties will contribute to endometriosis therapies.

## Abbreviations

CAR-M: macrophages with a chimeric antigen receptor
CAR-T: T-cell with a chimeric antigen receptor
BMDMs: bone marrow-derived macrophages
FBS: fetal bovine serum
H&E: hematoxylin and eosin
IHC: immunohistochemistry
LPS: lipopolysaccharides
NK cells: natural killer
PBS: phosphate-salt buffer solution
TCM: telocyte-conditioned medium
TRMs: tissue-resident macrophages

## 1 Introduction

Endometriosis is a serious gynecologic chronic inflammatory disease characterized by the growth of ectopic endometrial tissue outside the physiologic region [1]. According to WHO, this disease affects about 10% of women of reproductive age worldwide. This pathology causes severe pelvic pain and can also lead to infertility [2].

Three distinct types of endometriosis have been identified, each exhibiting unique characteristics regarding localization and pathophysiology. These include superficial peritoneal endometriosis, ovarian endometrioma, and deep infiltrating endometriosis. For the treatment of superficial peritoneal endometriosis, approaches such as laparoscopic removal of lesions, which is considered the gold standard, and drug therapy are available. Drug therapy can also be considered as prophylaxis to prevent the development of the other two types of endometriosis. At the same time, different surgical approaches are more typical for the treatment of ovarian endometrioma and deep infiltrating endometriosis [3].

The medications used to limit disease progression include combined oral contraceptives, nonsteroidal anti-inflammatory drugs, gonadotropin-releasing hormone receptor antagonists and agonists, progestins, and aromatase inhibitors [4,5]. These drugs primarily aim to reduce the pain associated with the disease [6,7]. Although these methods do not entirely eliminate foci, it is important to note that drug therapy can have severe side effects, including headaches, hot flashes, and decreased bone density, which increases the risk of fractures. It is also important to note that drug therapy only has a temporary effect, meaning that the results of therapy can only be observed during the course of medication. Additionally, taking medication during therapy prevents the possibility of pregnancy [6,7].

The previously mentioned therapeutic modality such as laparoscopic excision of ectopic foci is the gold standard in endometriosis therapy to date [8]. This therapeutic option can be prescribed when the patient does not respond to medical therapy [9]. Laparoscopic excision has been shown to reduce pain, but more than 22% of patients require re-operation [10]. In addition, surgery often requires extensive dissection, which can lead to postoperative complications; and it does not always resolve the problem of disease-related infertility [8,9].

Therefore, the development of new effective methods of minimally invasive therapy of endometriosis is required [11]. One of the most promising avenues in the treatment of endometriosis is currently cell therapy. The history of the development of this field dates to the 19th century. To date, cell therapy has been at the forefront of the development of therapeutic methods in various fields of medicine [12]. In particular, over 19,500 clinical trials have been registered to investigate the efficacy of therapeutic approaches for various diseases, including those pertaining to regenerative medicine, tumor therapy, and immunotherapy [12,13]. In a previous study, we evaluated potential trends in cell therapy for endometriosis. These therapeutic trends were considered to include suppression of estrogen-dependent growth, recruitment of stem cells to foci, angiogenesis in foci, resolution of fibrosis, and alteration of intracellular and extracellular signaling, as well as platelet depletion. Furthermore, current research in the field of endometriosis treatment is focused on the introduction of activated natural killer (NK) cells and macrophages, as well as the replacement therapy of ectopic foci with progesterone-sensitive endometrial stromal cells [14].

Macrophages are known to be leukocytes involved in innate and adaptive immunity. The functions of these cellular agents are extensive and include both recognition and neutralization of pathogens, initiation and resolution of inflammation, maintenance of tissue homeostasis and remodeling [15,16]. Macrophages can be divided into two categories: tissue-resident macrophages (TRMs) and bone marrow-derived macrophages (BMDMs). BMDMs differentiate from monocytes exiting a blood vessel into tissue [17]. TRMs are responsible for maintaining tissue homeostasis. Examples of their functioning include the removal of excess bone tissue by osteoclasts, the maintenance of vascular networks in various organs by TRMs, the removal of damaged cells, and the formation of the first line of defense against pathogens [18] They accomplish this through a number of mechanisms, including their impressive ability to chemotaxis, phagocytosis, secretion of inflammatory and anti-inflammatory cytokines, production of reactive oxygen and nitrogen species, and presentation of antigens to attract other immune cells [19].

While TRMs contribute to maintaining tissue homeostasis under physiological conditions, they can die and be replaced by BMDMs in response to signals from the lesion when the tissue is damaged. However, the activation of macrophages from monocytes arriving at the lesion does not correspond to the activation observed under physiological conditions. This ultimately results in the development of complications associated with the disease, which are mediated by macrophages. In this context, these BMDMs may be a promising target for the development of targeted therapies for various diseases [18].

In 2000, Charles D. Mills et al. developed a concept that proposed to define macrophages with pro- and anti-inflammatory phenotypes as M1 and M2 macrophages, respectively [20]. However, this paradigm is currently under serious criticism because it greatly simplifies the concept of existing phenotypes of macrophages, the transition between these phenotypes and the corresponding functions of macrophages [21]. Thus, the concept of M1/M2 macrophages does not allow to describe accurately enough the phenotype of macrophages involved in the pathogenesis of diseases and in the maintenance of homeostasis in tissues, since cells can express both M1 and M2 macrophage marker genes [22,23].

At the same time, the M1/M2 macrophage paradigm is convenient for application in *in vivo* studies. The phenotype of macrophages is induced by their microenvironment in the tissue, i.e. by the complex effects of multiple cytokines. Consequently, such a signal can summarily stimulate either proinflammatory or anti-inflammatory polarization [24]. Since our work is an *in vivo* study for which it is common to apply the M1/M2 paradigm, we will also use this concept. In addition, it has been shown that M2 macrophages are subdivided into M2a, M2b, M2c, and M2d, which differ in surface markers and biological functions [25]. And M1 macrophages are characterized by subdivision into M1a and M1b [26]. In this case, we analyzed the extreme forms of M1 and M2 macrophages in our study.

Macrophage-based cell therapy for various diseases is an emerging field. The current clinical trials devoted to the study of macrophages as potential agents are divided into five groups, each of which is focused on a distinct mechanism of action on cells. These mechanisms include the use of modified macrophages derived from induced pluripotent cells, the use of macrophages as a carrier of nanoparticles within themselves, as well as a carrier of substances on their surface [27]. The ability of macrophages to absorb and transport substances to the lesion focus represents a potential avenue for the delivery of therapeutic drugs. The fourth area of research into the therapeutic abilities of macrophages is the successfully developed technology for producing macrophages with a chimeric antigen receptor (CAR-M) that targets tumor cells and has phagocytic activity [28,29]. The fifth line of research is devoted to ex vivo reprogramming of patient macrophages and their subsequent injection into the patient [27].

In the case of administration of autologous reprogrammed macrophages, macrophages derived from the patient's peripheral blood monocytes, i.e., BMDMs, are mainly implied. In this case, peritoneal macrophages can also be used to develop therapeutic staging strategies. Peritoneal macrophages were found to be more advanced in development compared to BMDMs based on their structure and surface molecular characteristics. BMDMs exhibited the highest capacity for both cell growth and phagocytosis among the two types of macrophages. BMDMs secreted high amounts of suppressive cytokines (IL-10 and TGF-β). In few works [30,31] has been shown that BMDMs and PMs are not interchangeable, and the choice of cell type depends on the specific inquiries. BMDMs offer a broader range of responses in most situations, especially regarding phagocytosis, gene regulation, and secretion, making them suitable for cell biological inquiries. On the other hand, peritoneal macrophages are valuable for assessing the innate immune system's responsiveness within the context of the animal they originate from, particularly beneficial for knockout or genetically modified animals [31]. As an illustration, Dong et al. engineered CAR-M from peritoneal cells of gastric cancer patients by targeting them against tumor cells carrying human epidermal growth factor receptor 2 (HER2). The modified macrophages demonstrated antitumor effects in a mouse model of gastric cancer [32]. Thus, the object for cell therapy must, of course, be selected in relation to the goals set.

In our previous work, we performed a comparative analysis of the characteristics of tumor growth and endometriosis and found that these pathologies share many similarities. One such feature was the predominance of macrophages with an anti-inflammatory (M2) phenotype in progressive lesions. In addition to macrophages resident in endometriosis foci, peritoneal macrophages infiltrating ectopic lesions play an active role in the pathogenesis of the disease [33]. The discovery of this property of macrophages has led to the current focus on macrophages as a potential target or agent for endometriosis therapy. It should be noted, however, that research in this area is relatively scarce. So, in a study by Li et al., it was demonstrated that nanovesicles derived from M1 macrophages reduce the migration and invasion of endometrial stromal cells. Furthermore, in an *in vivo* model of endometriosis, it was shown that these nanovesicles cause peritoneal macrophages to polarize towards M1 and lead to a reduction in the size but not the number of lesions [34]. In our study, we decided to take a different approach.

M1 macrophages are known to have anti-tumor activity [35]. Therefore, we decided to investigate the potential anti-endometriosis properties of M1 macrophages in a syngeneic mouse model of endometriosis.

## 2 Material and methods

### 2.1 Animals

160 sexually mature female mice of the C57Bl/6 strain weighing 18–20 g. Specified number of animals includes animals used only for development and characterization of the endometriosis model (n = 70) and animals that were treated with cells at least three times (n = 90). The mice were obtained from the Stolbovaya laboratory animal nursery (Moscow region, Russia). Mice were maintained under standard conditions with free access to water and food, and a 12-h day/night cycle.

### 2.2 Syngeneic mouse model of endometriosis

The methodology of the resulting model is based on a protocol adapted from Mishra A. et al., 2020 [36]. This protocol was adapted by us for this study. On the first day, ovariectomy was performed in all animals under anesthesia with Zoletil 100 (Virbac, France) and Xylanite (Nita-Pharm, Russia) solution at 1.7 mg/mouse and 0.75 mg/mouse, respectively (mouse weight - 25 g). The mice were then subjected to chronic administration of 17*β*-estradiol (Sigma-Aldrich, USA) (300 ng per week) for two weeks.

Two weeks after ovariectomy, 80 mice were used as donors. They were euthanized with an overdose of anesthesia and their uterus was extracted. The extracted uterus was washed in sterile phosphate-buffered saline (PBS) containing 25000 units of penicillin and 25000 units of streptomycin and fragmented. Uterine fragments were injected intraperitoneally into 80 recipient mice. In 3 weeks after transplantation against the background of chronic administration of increased doses of estradiol, formed foci of endometriosis were detected in the abdominal cavity of mice [Approved by the local ethics committee of FSBSI “Petrovsky National Research Center of Surgery”, Protocol No. 3 dated March 23, 2023].

### 2.3 Non-invasive *in vivo* imaging of endometriosis foci

Prior to transplantation, uterine fragments were stained with membrane dye Cy7.5 for non-invasive detection of formed endometriosis foci after 3 weeks. A solution of 5 nM Cy7.5 dye was injected into the uterine fragments through an insulin syringe with a 23G needle. Then the uterine fragments were incubated in the same solution for 30 min at +37°C and then transplanted intraperitoneally into recipient mice.

We detected the foci on the IVIS® Spectrum In Vivo Imaging System (PerkinElmer, Inc., USA) 3 weeks after transplantation. Endometriosis foci were detected by fluorescence excitation (excitation filter - 745 ± 30 nm, emission filter - 800 ± 20 nm).

### 2.4 Histology and immunohistochemistry (IHC) of endometriosis foci before and after therapy

The formed endometriosis foci and uterus were extracted and fixed with liquid nitrogen and stored at -80 °C. Cryosections of 7–8 μm thickness were prepared and transferred to Superfrost Plus microscope slides (Thermo Fisher Scientific, USA).

Before staining, sections were washed in PBS for 5 min at room temperature. For overview histology of endometriosis foci, uterus, liver, spleen the sections were stained with hematoxylin and eosin (H&E). And rabbit specific HRP/DAB detection IHC kit (ab64261, Abcam, UK) was used for IHC analysis of foci and endometrium. The assay detected antigens by incubating sections with the following primary antibodies: goat anti-E-cadherin antibody (ab76319, Abcam, UK), rabbit anti-CD86 antibody (sc-9092, Santa Cruz Biotechnology, USA), rabbit anti-mannose receptor (CD206) antibody (ab64693, Abcam, UK), at a dilution of 1/200 each, overnight at +4 °C. After incubation with primary antibodies, the sections were washed in PBS and incubated with either anti-goat IgG HRP antibody solution (1/200) for 1 h at room temperature or with reagents from the rabbit specific HRP/DAB kit. Peroxidase was detected using 3,3′-diaminobenzidine solution (DAB chromogen). Cell nuclei were stained with hematoxylin.

Quantitative analysis of the content of positively stained cells was performed using QuPath software (UK) [37].

### 2.5 Real-time PCR of uterus, endometriosis foci before and after therapy, and RAW264.7 macrophages

RNA isolation from tissues and macrophages of RAW264.7 line was performed using ExtractRNA buffer (Eurogen, Russia) by guanidine thiocyanate-phenol-chloroform extraction. After extraction, cDNA was synthesized on the mRNA matrix using the Reverse Transcription Reagent Kit with MMLV Revertase (SK021) (Evrogen, Russia). Gene expression in the uterus and foci was detected using the following primers (forward/reverse) *GAPDH* (‘AGGCCGGTGCTGAGTATGTC/TGCCTGCTTCACCACCTTCT’), *SOCS3* (‘GCTCCAAAAGCGAGTACCAGC/AGTAGAATCCGCTCTCCTGCAG’), *TGFB1 (‘GTACTCTGGCAGTGACCCCG/AACTGCTCCACCTTGGGCTT’), IL-10 (‘GATGGGAGGGGTTCTTCCTTG/GGGATGACAGTAGGGGAACC’), IL12B (‘ATGAGGAGCTGGCTTTGGTC/TTGCATCCATTTGTGTGGCG’), TNF (‘TGGGACTGTTGCTAGCCCTG/ATGAGCCAGACCCACCTTGC’), IL23A (‘GCTGTGCCTAGGAGTAGCAG/CAGACCTTGGCGGATCCTTT’), ARG1 (‘ACGGCAGTGGCTTTAACCTT/AGGTAGTCAGTCCCTGGCTT’), IFNG (‘CTTCAGCAACAGCAAGGCGA/TCTGTCTGCAGTGGGGAAACA’), CCR7 (‘TCTTCCTCTCCATCCCGGAG/AGACTACCACCACGGCAA’).* M1 marker gene expression in RAW264.7 macrophages was detected using the following primers (forward/reverse): *NOS2 (‘GCTGCCAGGGTCACAACTT/CCTCACATACTGTGGACGGG’).* Amplification reactions were performed in triplicate in a DTprime DT96 detection amplifier (DNA-Technology, Russia). The level of relative gene expression was counted using QGENE software (2-ΔCt method) [38] with normalization of each gene expression to the GAPDH gene.

### 2.6 Western blot analysis of uterus, endometriosis foci before and after therapy, macrophage line RAW264.7

Western blot analysis was performed according to the protocol described in a previous study [39]. The following primary antibodies were used in the assay: mouse anti-GAPDH (Cat. No. 5G4, clone 4G5, HyTest, Russia) (1/5000), goat anti-arginase 1 (sc-18351, Santa Cruz, USA) (1/700), mouse anti-CD86 (ab213044, Abcam, UK) (1/2000), mouse anti-CD1 63 (sc-58965, Santa Cruz, USA) (1/2000), mouse anti-SOCS3 (ab236519, Abcam, UK) (1/2000), rabbit anti-CD206 (ab64693, Abcam, UK) (1/1000), rabbit anti-MARCO (ab256822, Abcam, UK) (1/1000); and the following secondary antibodies Goat anti-rabbit IgG H&L (HRP) (ab6721, Abcam, UK) (1/5000), Goat anti-mouse IgG H&L (HRP) (ab6789, Abcam, UK) (1/2000), Donkey anti-goat IgG (H + L) HRP (A15999, Invitrogen, USA) (1/2000). Membranes were developed using Clarity Western ECL Substrate (Biorad, USA). Signal was detected on a ChemiDoc Imaging System (Biorad, USA) using Image Lab Touch Software (Biorad, USA). The amount of protein content in the sample was estimated by normalizing to the signal of the loading control protein GAPDH.

### 2.7 Cell line

Mouse macrophage cell line RAW264.7 (Cat. No. 186, BioCollection, Moscow, Russia) was cultured in complete RPMI 1640 nutrient medium with glutamine (PanEco, Russia) supplemented with fetal bovine serum (FBS) (NuClopetm, USA), penicillin/streptomycin (PanEco, Russia) at +37°C and 5% CO2 in 175 cm^2^ culture flasks (Corning Inc., USA) until the cells reached 100% confluent monolayer.

### 2.8 Polarization of macrophages to the M1 phenotype

To obtain M1-polarized macrophages, cells were incubated with lipopolysaccharides from Escherichia coli O111:B4 (LPS) (Sigma-Aldrich, USA) (100 ng/mL) for 24h. Polarization of macrophages was confirmed by real-time PCR and Western blot analysis. Non-polarized macrophages cultured in nutrient medium were used as a polarization control.

### 2.9 Intraperitoneal injection of M1 macrophages in a mouse model of endometriosis

After polarization, M1 and control macrophages were stained with PKH26 Red Fluorescent Cell Linker Kit for General Cell Membrane Labeling (Sigma-Aldrich Co., USA) according to the manufacturer's protocol.

The endometriosis model animals were then divided into two groups, experimental and control, with 10 animals in each group. In experimental animals, M1-labeled macrophages (5 million cells in 300 μL) were injected intraperitoneally through a 29-gauge needle using an insulin syringe. Control animals were injected intraperitoneally with non-polarized labeled macrophages. Potential anti-endometriosis properties were analyzed 2 weeks after cell injection.

### 2.10 Flow cytometry of peritoneal macrophages

Peritoneal macrophages were collected from the peritoneal cavity, precipitated by centrifugation at 300*g* for 5 min at room temperature, lysed with red blood cell lysis solution (Miltenyi Biotec, 130-094-183, Germany) and fixed in an Inside Stain Kit (MACS, 130-090-473, Germany). After fixation, the cells were washed and resuspended in autoMACS® Rinsing Solution (Miltenyi Biotec, Germany).

To determine the presence of injected PKH26+ cells or cells resulting from cell division in the peritoneal fluid, the cell suspension was analyzed on a MACSQuant® Analyzer 10 flow cytometer (Miltenyi Biotec, Germany) using MACSQuantify™ Software 2.11.

To determine the phenotype of peritoneal macrophages before and after therapy, cells (cell suspensions were stained after therapy) were stained with a panel of anti-mouse antibodies for 1 h at room temperature in the dark: Ly-6C-PE REAfinity™ (130-111-778, Miltenyi Biotec, Germany), CD11b-Vio®Bright FITC REAlfinity™ (130-113-243, Miltenyi Biotec, Germany), CD86-PE-Vio®770 (130-105-135, Miltenyi Biotec, Germany), MHC Class II-PerCP-Vio® 700 (130-103-875, Miltenyi Biotec, Germany), F4/80-PE REAfinity™ (130-102-422, Miltenyi Biotec, Germany), F4/80-PerCP-Vio® 700 REAfinity™ (130-118-327, Miltenyi Biotec, Germany), CD206-PE (12206182, eBioscience™, USA), CD163-PE (2006963, eBioscience™, USA).

The phenotype of peritoneal macrophages and the detection of PKH26+ cells in peritoneal fluid were analyzed by flow cytofluorometry and the data were processed by Flowing software (Turku Centre for Biotechnology, Finland).

### 2.11 Statistical analysis

Statistical processing of the data was performed using the GraphPad Prism 8 program (GraphPad Software, USA). Normality of distribution was determined by Shapiro-Wilk test. For normal distribution, the *t*-test for pairwise comparisons or one-way ANOVA for multiple comparisons with post hoc Tukey's test was applied to the data. For non-normal distribution or distribution with small sample size, Mann-Whitney test for pairwise comparison and Kraskell-Wallis test for multiple comparison with post-hoc Dunn's test were used. Two-tailed tests and 95% confidence intervals were used in the analysis. Differences were considered statistically significant at p<0.05.

## 3 Results

### 3.1 Endometriosis foci formed in a syngeneic mouse model

**Fig. 1:**
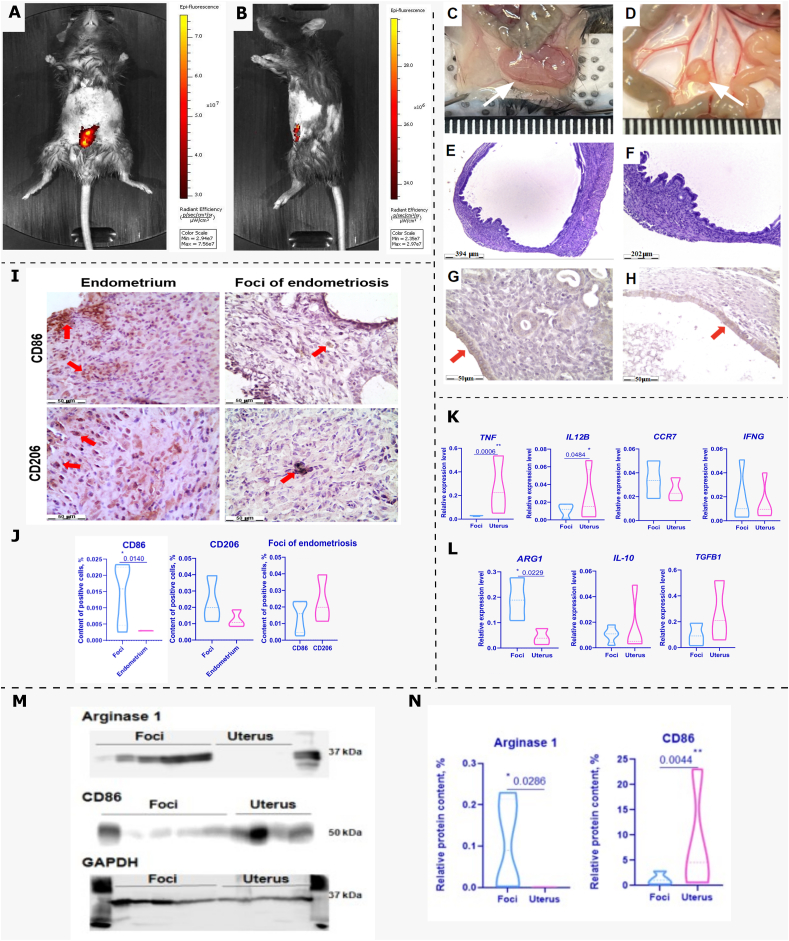
**Investigation of the macrophage phenotype in the formed foci of a syngeneic mouse model of endometriosis.A, B** – In vivo live imaging of Cy7.5-labeled endometriosis foci formed in mice 3 weeks after transplantation: abdominal view – **A**, lateral view – **B**. **C, D** – Image of formed endometriosis foci in mice. White arrows indicate foci. The scale value is 1 mm. **E, F** – Section of an endometriosis foci stained with H&E. Scale bar C – 394 ***μ***m, D – 202 ***μ***m. **G, H** – Detection of E-cadherin by the HRP DAB substrate in epithelial cells of endometrium and of endometriosis foci, respectively. Nuclei are stained with hematoxylin. Red arrows indicate positively stained cells. Scale bar 50 ***μ***m. **I** – Detection of CD86+ and CD206+ macrophages by HRP DAB substrate in endometrium and in foci of endometriosis. Nuclei are stained with hematoxylin. Red arrows indicate positively stained cells. Scale bar 50 ***μ***m. **J** – Percentage of CD86+ and CD206+ macrophages in endometriosis foci compared to endometrium. **K, L** – Relative expression levels of M1 macrophage marker genes **(K)** and M2 macrophage marker genes **(L)** in endometriosis foci and in uterus. Expression levels were calculated using the 2-ΔCt method. The expression of each gene was normalized to the housekeeping gene GAPDH. **M** – Western blot analysis of Arginase 1, CD86 proteins in endometriosis foci and in uterus. Loading control GAPDH. **N** – Relative level of analyzed proteins in endometriosis foci and in uterus. Statistical analysis was carried out by non-parametric Mann–Whitney *U* test, *–р-value<0,05, **– p-value<0,005.

As a result of intraperitoneal injection of uterine fragments with thickened endometrium from donor mice into recipient mice, we detected formed endometriosis foci after 3 weeks. We non-invasively visualized the formation of endometriosis foci using Cy7.5 fluorescence detection on the IVIS® Spectrum In Vivo Imaging System (Fig. 1A and B) (S.1). We then observed formed foci at autopsy, which were pink, yellow, or yellow with black splashes, thin-walled cysts penetrated by blood vessels and attached to the peritoneum or mesentery (Fig. 1C and D) (S.2).

### 3.2 Histology of the obtained foci

In histologic analysis of the formed foci of murine endometriosis, we found that the cyst cavity was surrounded by a layer of prismatic epithelium, and the wall contained stromal cells, glands, blood vessels, and a layer of muscle cells surrounding the foci on the outside (Fig. 1E and F). Epithelial cells in the foci were detected by IHC staining for E-cadherin, which is also produced by mouse endometrial epithelial cells and is characteristic of mouse endometrial epithelial cells (Fig. 1G and H).

### 3.3 Predominance of M2 macrophages over M1 macrophages in endometriosis foci

In IHC analysis, we found that CD86+ and CD206+ cells were present in the foci of endometriosis and in the endometrium (Fig. 1 I). Based on their localization and morphology, the observed CD86+ and CD206+ cells are probably macrophages. When quantitative analysis of these macrophages was performed, we found no significant differences in the content of CD86+ and CD206+ macrophages in the foci of endometriosis (Fig. 1 J). In our study, we assumed that CD86+ macrophages are M1 macrophages and CD206+ macrophages are M2 macrophages. Thus, by IHC analysis, we found that both M1 and M2 macrophages were present in the endometriosis foci.

We then performed real-time PCR on endometriosis foci to determine the expression of M1 and M2 macrophage marker genes. Upon analysis, we found significant differences in the expression levels of M1 macrophage marker genes TNF, IL12B (p-value<0.005 and p-value<0.05, respectively) with predominant expression of these genes in the uterus, which we used as an endometrial tissue control. In the case of IFNG and CCR7, we observed comparable expression levels of these genes in the foci and in the uterus (Fig. 1 K). The analysis of M2 marker gene expression revealed significant differences in the expression levels of ARG1 (p-value <0.05), with a predominant presence in the endometriosis foci (Fig. 1 L). In the case of IL-10 and TGFB1 genes, comparable expression levels were observed in the foci and uterus.

Thus, we were able to show that the expression of the M1 macrophage marker genes TNF and IL12B was significantly decreased in the endometriosis foci, and the expression of the M2 macrophage marker gene ARG1 was significantly increased.

In addition to the real-time PCR data, we also showed a significant increase (p-value<0.05) in the production of the protein encoded by the *ARG1* gene, arginase 1, in the endometriosis foci compared to the uterus (Fig. 1K and L) (S.3). In addition, the production of the M1 marker of macrophages, CD86, was significantly increased in the uterus (p-value<0.005) compared to the production of this protein in the foci.

### 3.4 Therapy of animals with endometriosis with activated M1 macrophages

**Fig. 2:**
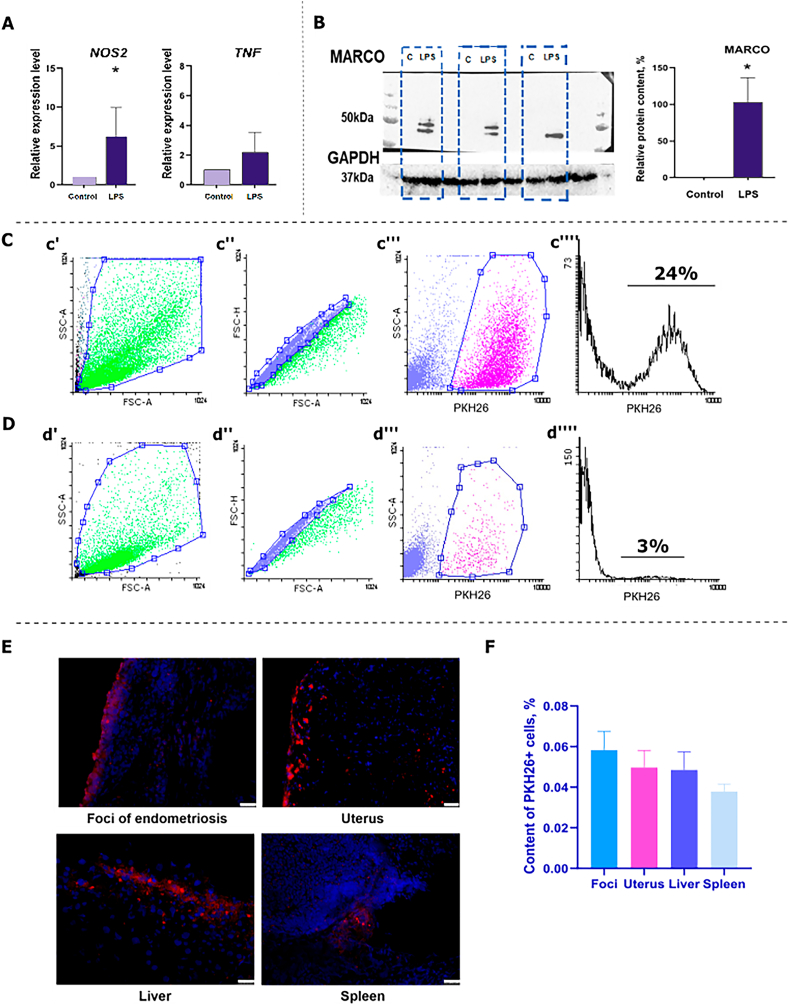
**Chemical reprogramming of macrophages toward the M1 phenotype and their introduction into a syngeneic model of endometriosis.А** – Relative expression level of NOS2 and TNF genes in RAW264.7 cell line. Control – cells incubated in complete nutrient medium. LPS – cells incubated in complete nutrient medium supplemented with LPS (100 ng/mL). Expression levels were calculated as indicated in Fig. 1**I and J**. **B** – Western blot analysis of MARCO proteins in the RAW264.7 cells (Control, LPS). Loading control GAPDH. Statistical analysis **(A, B)** was carried out by non-parametric Mann-Whitney *U* test, *-p-value<0.05. **C, D** — Gating strategy in the analysis of peritoneal macrophages from mice with injected M1 macrophages and with injected control macrophages, respectively: c', d'- FSC-A–SSC–A dot-plot, the region of the main cell pool is highlighted; c'', d''- FSC-A–FSC–H dot-plot, the singlet region is highlighted; **c''', d'''**- PKH26-SSC-A dot-plot, the pool of PKH26+ cells is highlighted; **c'''', d''''**- histogram of PKH26 fluorescence signal intensity distribution. **E** – Detection of PKH26+ cells in endometriosis foci, uterus, liver and spleen. Red is PKH26, blue is DAPI. **F** – Percentage of PKH26+ cells in foci of endometriosis, uterus, liver and spleen. Statistical analysis was carried out by One-way ANOVA with Tukey's post hoc tests.

The RAW264.7 macrophages were incubated with LPS (100 ng/mL) to activate them towards the M1 phenotype. The NOS2 gene expression level was significantly increased (p-value <0.05) after macrophages were polarized compared to control macrophages. TNF expression levels were comparable between control and reprogrammed cells (Fig. 2 A). Furthermore, we observed a significant increase in the production of the M1 macrophage marker MARCO in the cells upon successful activation of macrophage polarization towards the M1 phenotype (Fig. 2 B) (S.3).

Following successful polarization, we labeled the cells with Red Fluorescent Cell Linker PKH26 and administered 5*10^6 cells/mouse intraperitoneally to mice with endometriosis. As a control therapy, we injected unpolarized PKH26-labeled RAW264.7 cell line RAW264.7 cells. These findings demonstrate the clear superiority of reprogrammed macrophages over non-polarized macrophages. The results were clear: after two weeks, we detected 24 % PKH26+ macrophages in the peritoneal fluid of animals injected with reprogrammed macrophages (Fig. 2C), while only 3 % PKH26+ macrophages were present in the peritoneal fluid of mice injected with non-polarized macrophages (Fig. 2 D).

Endometriosis foci, uterus, liver, and spleen were analyzed in mice after treatment to determine the presence of PKH26+ cells. The investigation revealed the migration pathways of injected cells as well as their possible clones. PKH26+ cells were present in all analyzed tissues two weeks after injection of M1-labeled macrophages (Fig. 2E). The number of PKH26+ macrophages in endometriosis foci was higher than in the uterus, liver, and spleen. However, the difference was not statistically significant (Fig. 2F). In contrast, no PKH26+ labeled cells were detected in the analyzed tissues of animals injected with labeled control cells.

Also, we compared the histology of liver, spleen, and uterus by H&E staining for inflammation. We saw that histologically all tissues did not differ between healthy animals, animals with endometriosis and animals injected with M1 macrophages (S.4). We did not see foci of inflammation or tissue necrosis. At the same time, the liver in all groups of animals was characterized by the presence of a small lymphatic follicle in the perivascular area. Thus, injected M1 macrophages do not have a toxic effect on the liver, uterus, and spleen.

### 3.5 Observed anti-endometriosis properties of M1 macrophages

**Fig. 3:**
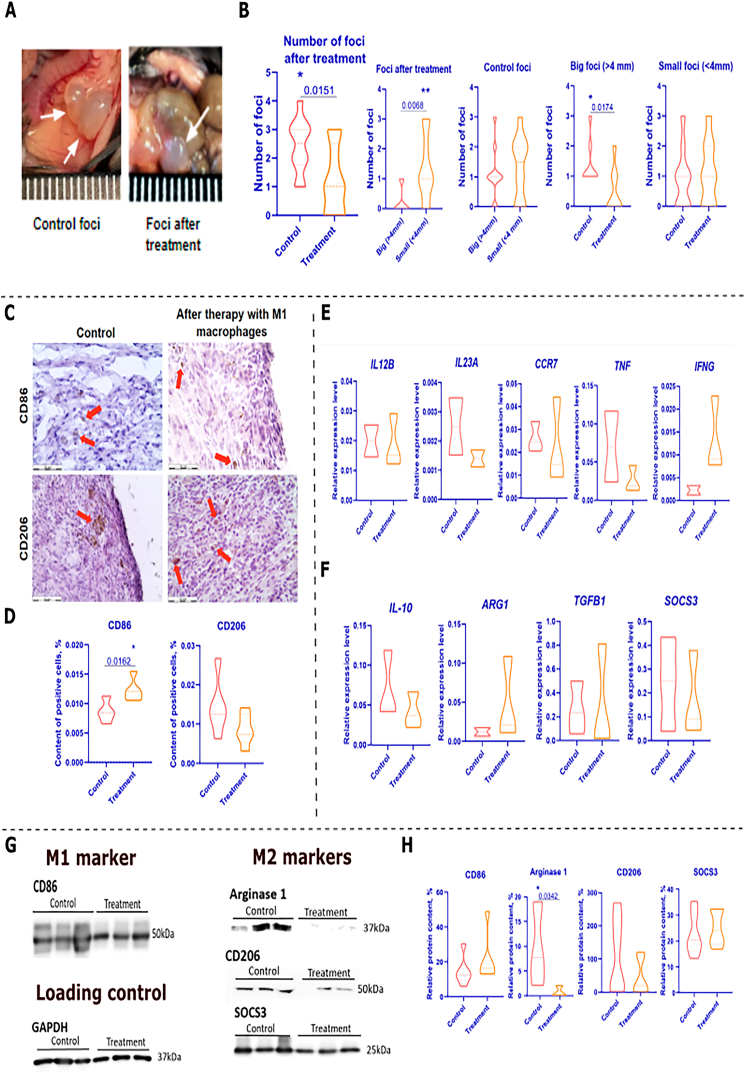
**Analyzing the results of introducing M1 macrophages into a syngeneic model of endometriosis. А** – Image of endometriosis foci in control mice and in mice after M1 macrophage therapy. White arrows indicate the foci. The scale value is 1 mm. **B** – Estimation of the number of the foci in mice after treatment compared with control mice. **C** – Detection of CD86+ and CD206+ macrophages by HRP DAB substrate in endometriosis foci. Nuclei are stained with hematoxylin. Red arrows indicate positively stained cells. The scale bar is 50 ***μ***m. **D** – Percentages of CD86+ and CD206+ macrophages in the endometriosis foci. **E, F** – Relative expression levels in the endometriosis foci and the uterus of M1 macrophage **(E)** and M2 macrophage **(F)** marker genes. Expression levels were calculated as indicated in Fig. 1**I and J**. **G** – Western blot analysis of Arginase 1, CD206, SOCS3, CD86 proteins in endometriosis foci, control and after M1 macrophage therapy. Loading control GAPDH. **H** – Relative level of analyzed proteins in the endometriosis foci. Statistical analysis was carried out by non-parametric Mann–Whitney *U* test, *–р-value<0,05, **– p-value<0,005.

Mice treated with M1 macrophages showed a reduction in lesion size compared to the control group after 14 days of treatment (Fig. 3A).

Previous studies have categorized lesions into groups based on size. In the case of examining potential prognosis in gastric cancer, tumors were classified as either ‘small’ or ‘large’. For ‘early gastric cancer’, tumors were categorized as smaller than 3 cm or larger than 3 cm. For advanced gastric cancer tumors, they can be classified as small (less than 6 cm) or large (6 cm or more) [40]. In this study, ectopic lesions were classified as small foci (less than 4 mm) or large foci (4 mm or more) based on their size.

M1 macrophage therapy resulted in a significant reduction in the number of lesions (p-value <0.05) and ‘big foci’ compared to the control group (Fig. 3B). The distribution of ‘big foci’ and ‘small foci’ was compared, and it was observed that ‘big foci’ were predominantly present in the control animals. This difference was statistically significant (p-value <0.05) when compared to the experimental group.

The phenotype of macrophages in the endometriosis foci after therapy was subsequently analyzed in comparison to the control foci. Through IHC analysis, the presence of CD86+ and CD206+ macrophages were detected (Fig. 3C). The study confidently reports a significant increase (p-value<0.05) in CD86+ macrophage content in the foci after M1 macrophage therapy compared to control foci (Fig. 3 D). Although CD206+ macrophage content decreased in the foci after therapy, the difference with control foci was not statistically significant. M1 macrophage therapy shifts the balance in endometriosis foci towards the predominance of CD86+ macrophages over CD206+ macrophages, resulting in an increase in macrophages with proinflammatory properties.

Real-time PCR analysis of the foci revealed the presence of macrophages with both M1 and M2 phenotypes in both control and post-treatment foci (Fig. 3E and F). The study found no significant differences in relative expression levels between the treatment and control groups.

However, Western blot analysis of foci revealed the production of M2 macrophage markers, including Arginase 1, CD206, and SOCS3, as well as the M1 macrophage marker CD86 (Fig. 3 G) (S.3). Additionally, a decrease in the production of the M2 macrophage marker Arginase 1 was found in the endometriosis foci after therapy compared to the control group (Fig. 3H) (p-value<0.05). Furthermore, therapy resulted in an increase in CD86 production and a decrease in CD206 and SOCS3 production in the foci. Although these differences were not statistically significant when compared to the foci from control animals.

### 3.6 Analysis of peritoneal macrophages infiltrating endometriosis foci

**Fig. 4:**
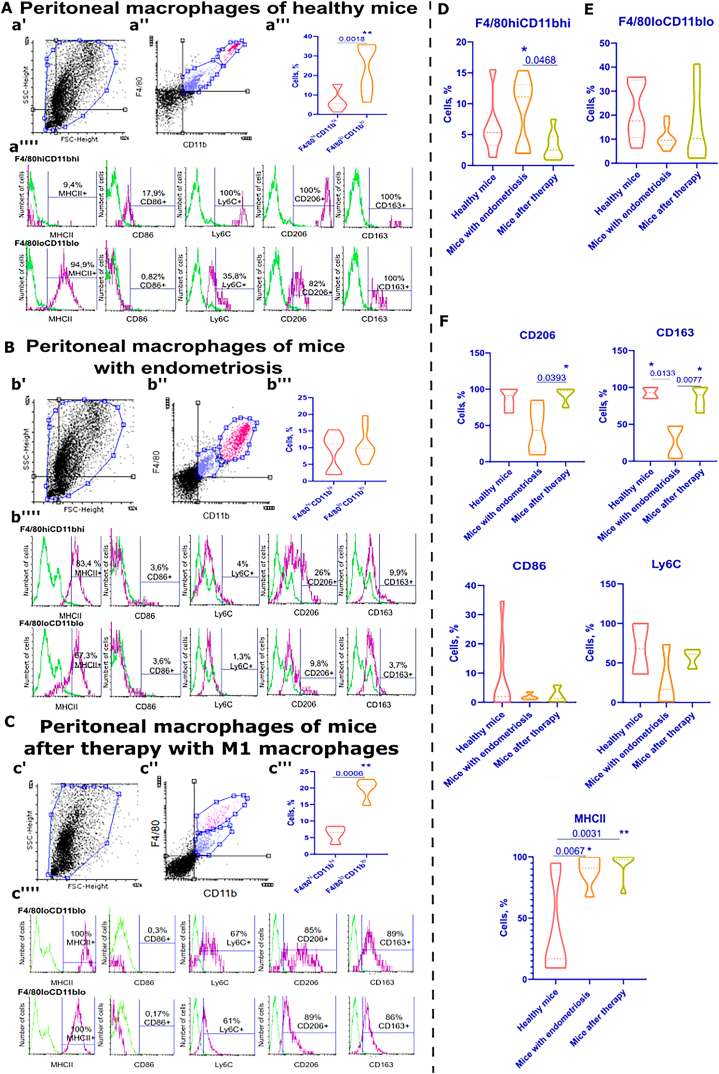
**Analysis of peritoneal macrophages from three groups of animals.** Healthy mice **(A)**, mice with endometriosis **(B)**, mice after M1 macrophage therapy **(C)**: **a', b', c'** – isolation of the main pool of cells on FSC-SSC dot-plot; **a'', b'', c''** – isolation of F4/80^hi^CD11b^hi^ (pink) and F4/80^lo^CD11b^lo^ (blue) subpopulations; **a''', b''', c''''** – percentage of F4/80^hi^CD11b^hi^ and F4/80^lo^CD11b^lo^ in the pool of peritoneal macrophages; **a'''', b'''', c''''** – histograms of fluorescence signal intensity distribution, detection of MHCII+, CD86+, Ly6C+, CD206+, CD163+ cells. Green contour is negative control, purple contour is stained sample. **D** – Percentage content of F4/80^hi^CD11b^hi^ in peritoneal fluid of three groups of mice. **E** − Percentage content of F4/80^lo^CD11b^lo^ in the peritoneal fluid of three groups of mice. **F** – Percentage of CD206+, CD163+, CD86+, MHCII+ and Ly6C+ cells in the peritoneal fluid of three groups of mice. Statistical analysis was carried out by non-parametric Mann–Whitney *U* test (**a’’’**, **b’’’**, **c’’’**) and One-way ANOVA with Tukey's post hoc tests (**D – F**), *–р-value<0,05, **– p-value<0,005.

The peritoneal macrophages infiltrating endometriosis foci were analyzed by isolating the main cell pool area on the dot-plot (Fig. 4 a', b', c'). Two subpopulations, F4/80^hi^CD11b^hi^ and F4/80^lo^CD11b^lo^, were distinguished (Fig. 4 a'', b'', c''). The percentage of these subpopulations in the peritoneal fluid of healthy mice, mice with endometriosis, and mice after M1 macrophage therapy was then evaluated (Fig. 4 a''', b''', c'''). Our analysis of peritoneal macrophages revealed a significant predominance of the F4/80^lo^CD11b^lo^ subpopulation over F4/80^hi^CD11b^hi^ in both healthy animals and animals after therapy (p-value<0.005 and p-value<0.05, respectively). Furthermore, mice with endometriosis exhibited comparable levels of F4/80^hi^CD11b^hi^ and F4/80^lo^CD11b^lo^ content. These findings demonstrate a clear and consistent pattern that supports our conclusions with confidence.

The subpopulations we identified, F4/80^hi^CD11b^hi^ and F4/80^lo^CD11b^lo^, were characterized by a low (9.4%) and high (94.9%) content of MHCII+ cells, respectively (Fig. 4 a''''). In animals with endometriosis and after therapy, both subpopulations showed an increased content of MHCII+ cells (Fig. 4 b'''', c'''').

The phenotypic analysis revealed that both healthy mice and mice after therapy had most macrophages producing CD206 and CD163 (Fig. 4 a'''', c''''). However, in mice with endometriosis, there was a decrease in the content of CD206+ and CD163+ macrophages in peritoneal fluid (Fig. 4 b''''). Furthermore, the F4/80^hi^CD11b^hi^ subpopulation of healthy animals had a small percentage of CD86+ macrophages (17.9%), which were absent in the F4/80^lo^CD11b^lo^ subpopulation (Fig. 4 a''''). CD86+ macrophages were absent in mice with endometriosis after therapy, among both F4/80^hi^CD11b^hi^ (3.6% and 0.3%) and F4/80^lo^CD11b^lo^ (3.6% and 0.17%) (Fig. 4 b'''', c'''').

Peritoneal macrophages were also analyzed for their phenotype and the level of production of the monocyte marker Ly6C. Analysis revealed that most peritoneal macrophages in healthy mice and mice treated with M1 macrophages produced Ly6C. However, Ly6C+ cells were absent in the peritoneal fluid of animals with endometriosis (Fig. 4 a'''', b'''', c'''').

A comparative analysis of subpopulations among the three groups of animals showed similar levels of F4/80^lo^CD11b^lo^ content in healthy mice and mice after therapy (Fig. 4 E). Meanwhile, the proportion of F4/80^hi^CD11b^hi^ content increases in animals with endometriosis, and there is a significant difference (p-value<0.05) in F4/80^hi^CD11b^hi^ content in animals after treatment (Fig. 4 D). Our analysis of the phenotypic profile of macrophages revealed some interesting findings. CD163+ cells showed a significant increase (p-value<0.05) in healthy mice and mice after therapy compared to mice with endometriosis. Furthermore, animals after therapy showed a significant increase in CD206+ cells compared to animals with endometriosis (Fig. 4 F). These results demonstrate the effectiveness of the therapy in altering the phenotypic profile of macrophages. In contrast, CD86+ cells were almost absent in the peritoneal fluid of mice with endometriosis and mice after therapy but were present in healthy mice. In both healthy mice and mice after therapy, there was a predominance of Ly6C+ cells compared to animals with endometriosis. However, there were no significant differences found between the groups. Notably, the production of MHCII by peritoneal macrophages was significantly increased in mice with endometriosis (p< 0.05) and after therapy (p< 0.005) compared to healthy mice.

## 4 Discussion

Macrophages have been shown to play a critical role in the development of endometriosis. Macrophages present in lesions represent a heterogeneous population including endometrial macrophages, infiltrating peritoneal macrophages and BMDMs [41]. It is known that endometrial macrophages do not cause the development of endometriosis foci, but only promote their growth. While BMDMs have a protective function in preventing the formation of ectopic lesions [42].

M1 macrophages have shown promising potential as cellular agents for therapy of various diseases, including malignant tumors, endometriosis, ischemic stroke, heart and renal failure, and neurodegenerative diseases [14,27,43]. This study aims to evaluate the anti-endometriosis properties of M1 macrophages in an *in vivo* syngeneic model of endometriosis. If this technology is translated to patients, then of course autologous cells should be used. The most suitable source of mouse macrophages for endometriosis treatment can be their bone marrow precursors or blood monocytes. Due to the difficulty of obtaining and the small volume of blood from mice, we chose the mouse macrophage cell line RAW264.7 as a cell therapeutic agent. This approach was also used in several other works [[44], [45], [46], [47]].

Our syngeneic mouse model of endometriosis has successfully demonstrated the formation of yellow, yellow-black, and pink cysts with a thin wall containing epithelial and stromal cells, glands, and blood vessels. The architecture of the foci is consistent with the literature, confirming the validity of our model. Mishra et al. previously demonstrated that the formed foci were transparent and contained blood vessels. Histological analysis revealed the presence of a stromal component, well-developed glands, and epithelial cells in the wall of endometriosis foci from a murine endometriosis model [36]. It is important to note that ectopic lesions in female patients exhibit varying morphologies. Endometriosis foci present as bluish-black or white scars, deep nodules, yellow patches, transparent, red, or brown cysts [48,49]. The different morphologies of endometriosis foci are associated with the stages of endometriosis development. Transparent cysts may be characteristic of the early stages of the disease, as the vessels have not yet sprouted strongly enough into the foci. Red foci may signify an advanced stage, and white scars may signify the fibrosis stage of the foci [49]. The endometriosis foci we obtained correspond to an intermediate stage between early and advanced endometriosis.

Our team has successfully demonstrated the possibility of non-invasive diagnosis of endometriosis foci using a live fluorescence imager. Our findings have shown that this method is a reliable and efficient way to detect fluorescent emission from the animal's body [50]. This method is a diagnostic tool like X-ray, magnetic resonance imaging, ultrasound diagnostics, and computed tomography. It offers real-time imaging, multiplex analysis, and minimal invasion into the body. However, its shallow penetration ability due to the absorption and scattering of excitation light in the tissue is a serious limitation. When selecting fluorophores, it is crucial to consider the organism's endogenous autofluorescence. In vivo imaging is an invaluable tool for preclinical animal studies, including the development of diagnostic and therapeutic methods for various diseases [51,52]. Our study utilized this method for non-invasive diagnosis of endometriosis foci. Fluorescence imaging, which is currently used in surgery to determine resection margins, has the potential to be implemented in other medical fields with further improvement of optical techniques and fluorescent probes [51,52].

CD86, TNF-α, iNOS, IL-12β, CCR7, IL-23a, MARCO, and IFN-ɣ are known to be markers of M1 macrophages, while CD206, Arginase 1, TGF-β, IL-10, and SOCS3 are specific markers of M2 macrophages [[53], [54], [55], [56], [57], [58], [59]]. Thus, macrophages expressing these markers can be confidently classified as either M1 or M2 macrophages. Both M1 and M2 macrophages were present in the ectopic lesions formed, with M2 macrophages predominating over M1 macrophages. This shift in macrophage balance in mouse and human ectopic endometrioid foci was also demonstrated by Bacci et al. in their study [60]. It is known that the phenotype of macrophages in ectopic lesions can be different depending on the stage of the disease, e.g. in the early stage of endometriosis development, macrophages with a proinflammatory phenotype predominate in the lesions, which then polarize towards an anti-inflammatory phenotype. Macrophages with an anti-inflammatory phenotype promote tissue repair, lesion growth, angiogenesis, and neurogenesis in foci [33,61]. In this regard, we can assume that the degree of endometriosis we observed in the animal model, as well as the morphology of the obtained endometriosis foci, is consistent with the development of endometriosis.

After therapy, we found that activated M1 macrophages persist and infiltrate endometriosis foci. The effect we observed of M1 macrophage migration into the peritoneal inflammatory lesion is consistent with literature data [15]. Indeed, in the previous research by Guo et al. investigating the possibility of using M1 macrophages as “anticancer drug carriers”, they showed that injected M1 macrophages derived from RAW264.7 macrophages polarized by LPS and loaded with doxorubicin accumulated in tumor lesions in a mouse model of ovarian carcinoma [15]. As a result of M1-polarized macrophage therapy, we observed a significant reduction in the number of endometriosis foci and their average size compared to control animals. Thus, in a previous study, the effect of telocyte-conditioned medium (TCM), which stimulates macrophage polarization toward the M1 phenotype, on endometriosis foci was evaluated. It was shown that TCM injection into the mouse model of endometriosis led to a decrease in the size of foci, suppression of angiogenesis in them, and suppression of lesion invasiveness. And TCM-activated M1 macrophages suppressed the production of VEGF and MMP9 in endometrial stromal cells [62]. In addition, the development of endometriosis is known to be promoted by weakened immune surveillance of ectopic lesions, which is a decrease in cytotoxic activity of NK cells, decreased phagocytosis by macrophages and neutrophils, which contributes to the maintenance of invasion and growth of ectopic lesions [63,64]. Therefore, we hypothesize that the administration of M1 macrophages may contribute to the suppression of foci angiogenesis and invasiveness and may potentially recruit and activate immune cells through cytokines and may potentially participate in phagocytosis of foci cells. This hypothesis is supported by current research, but further investigation is necessary to fully comprehend the molecular mechanisms of M1 macrophage influence on endometriosis foci.

Furthermore, we have excitingly demonstrated that M1 macrophage therapy effectively shifts the balance in endometriosis foci towards a predominance of M1 macrophages over M2 macrophages. These remarkable findings are consistent with a previous study where M1 macrophages associated with nanoparticles were peritumorally injected into a xenograft model of head and neck tumors [65]. The results of Im et al. showed enhanced tumor infiltration by these cells as a result of M1 macrophage injection [65].

Our analysis of peritoneal macrophages in healthy mice revealed that the isolated subpopulations of F4/80^hi^CD11b^hi^ and F4/80^lo^CD11b^lo^ were characterized by low and high levels of MHCII+ cells, respectively. In mice with endometriosis and after therapy, we observed a significant increase in the content of MHCII+ cells in peritoneal fluid in both subpopulations. It is important to note that MHCII is a molecule expressed on antigen-presenting cells that provides antigen presentation to CD4+ T-cells. This molecule has a dual function in macrophages. It maintains immune tolerance in the tissue and enhances MHCII production for antigen presentation when pathogens or foreign cells enter the tissue [66]. Our administration of syngeneic uterine fragments and the formation of endometriosis foci enhanced MHCII production by peritoneal macrophages, stimulating the development of an immune response. The introduction of RAW264.7 cells, which are foreign to the animal organism, may have also contributed to the increase in the content of MHCII+ cells in peritoneal fluid. Ly6C production levels in mouse peritoneal macrophages are frequently analyzed to distinguish between monocyte subtypes. Ly6C^hi^ monocytes are assumed to migrate from the bone marrow and infiltrate the focus of inflammation, while Ly6C^lo^ monocytes are believed to be the precursor of resident macrophages in tissues [67]. Our study shows that Ly6C+ macrophages disappear in the peritoneal fluid of animals with endometriosis, compared to healthy animals and animals after therapy. This is consistent with a previous study on macrophage infiltration in injured muscle, which demonstrated that Ly6C+ macrophages heavily infiltrate the focus at the early stage of injury, and at the late stage of tissue regeneration, Ly6C+ macrophages disappear and Ly6C- macrophages begin to predominate in the lesion [68]. In this regard, we suggest that the peritoneal macrophages we analyzed, which participated in the growth of foci, 3 weeks after intraperitoneal transplantation of uterine fragments show a similar phenotype with the stage of late tissue regeneration. We found that in healthy animals and in animals after therapy with M1 macrophages in peritoneal fluid there was an anti-inflammatory background all cells carried markers of M2 phenotype. This may indicate the absence of the acute phase of inflammation in the abdominal cavity in these animals [21]. In addition, anti-inflammatory macrophages disappeared from peritoneal fluid in animals with endometriosis.

Thus, the idea of our study of the anti-endometriosis potential of macrophages was based on the similarities between the development of tumor growth and endometriosis [33]. It is well known that macrophages can exert antitumor effects through several mechanisms. Primarily, macrophages can secrete cytokines that induce tumor cell death. These include TNFa, Il12, IL6, Il1b, and NO release [[69], [70], [71]]. The aforementioned cytokines are among the M1 markers of macrophages, which we utilized as a therapeutic agent for endometriosis treatment.

Another mechanism by which macrophages can eliminate tumor cells is the activation of effector immune cells, primarily NK cells, T-cytotoxic and B-lymphocytes. M1 macrophages [72] are also capable of activating various lymphocyte populations. Another mechanism of lymphocyte recruitment to the tumor or endometriosis focus is their attraction by chemokines. Among the markers of M1 macrophages are chemokines such as CCL5, CXCL9, and CXCL10 [59]. These factors have the capacity to attract a diverse range of leukocytes, including monocytes, lymphocytes, and various granulocytes. Accordingly, we propose the following mechanism for the anti-endometriosis activity of our injected macrophages. Macrophages (M1) injected into the abdominal cavity secrete a set of cytokines and chemokines, which cause cell death in the endometriosis focus and attract lymphocytes, monocytes, macrophages, and granulocytes to the focus. These cells are then activated by the cytokines secreted by the macrophages, including TNFa, Il12, IL6, and Il1b, which cause their proinflammatory anti-endometriosis activation.

Thus, macrophages represent promising agents for cell therapy of endometriosis in the future. It is worth noting that the use of macrophages in the clinic is still a developing field. While there are limitations to their use, extensive preclinical research can overcome these challenges. These are limitations such as the limitations of spontaneous polarization at the lesion focus, limited duration of activity, and the ability to mass-produce macrophages for therapy [27]. Regarding the adverse effects of M1 and M2 macrophage administration, no such data are available in the literature [70,73]. However, it is important to note that marked inflammatory reactions cannot be excluded, especially when administering M1 macrophages, which are observed in chimeric antigen receptor T-cells (CAR-T) therapy [74]. Furthermore, it is known that M1 polarized macrophages can perpetuate inflammation induced by granulosa cells in patients with endometriosis, which may contribute to reduced female fertility [75]. Therefore, much research is needed to overcome all these limitations.

A potential complication of M2 macrophage administration is the stimulation of endometriosis nidus development. M2 macrophages secrete factors that are like tumor-associated macrophages, which stimulate angiogenesis in the foci, its growth, and metastasis. This process has been observed in studies [27]. Another potential complication of introducing M2 macrophages into the abdominal cavity is the development of adhesions. M2 macrophages have been shown to stimulate the formation of intercellular substances [76].

In conclusion, the demonstrated anti-endometriosis activity of M1 polarized macrophages provides a strong basis for further *in vitro* and *in vivo* studies using human material. The promising prospect of introducing the administration of autologous polarized macrophages to patients with endometriosis into clinical practice highlights the potential for significant advancements in treatment.

## Funding

This work was supported by the 10.13039/501100012190Ministry of Science and Higher Education of the Russian Federation agreement No. 075-15-2022-294 dated April 15, 2022 to P.V. A part of the work concerning the mouse model was supported by State Assignment №123030700103–6 that was funded by the 10.13039/501100012190Ministry of Science and Higher Education of the Russian Federation to T.F. Also, the corresponding results were obtained with the financial support of the Russian Federation represented by the 10.13039/501100012190Ministry of Science and Higher Education of the Russian Federation agreement No. 075-15-2021-1356 (internal number of the Agreement 15.SIN.21.0011); (ID: RF 0951.61321X0012) dated October 7, 2021 to T.F.

## Ethics statement

This research was conducted in accordance with the U.K. Animals (Scientific Procedures) Act, 1986 and associated guidelines, the European Communities Council Directive 2010/63/EU, and the National Institutes of Health – Office of Laboratory Animal Welfare policies and laws. Also, this research was conducted in accordance with the ARRIVE guidelines. All experimental protocols approved by the local ethics committee of FSBSI “Petrovsky National Research Centre of Surgery” (protocol No. 3 of March 23, 2023).

## Data availability statement

The data associated with our study have not been deposited into a publicly available repository. The datasets supporting the conclusions of this article are included within the article and its Supplementary data.

## CRediT authorship contribution statement

**Daria Artemova:** Writing – review & editing, Writing – original draft, Visualization, Validation, Resources, Methodology, Investigation, Formal analysis, Data curation, Conceptualization. **Polina Vishnyakova:** Writing – review & editing, Supervision, Resources, Methodology, Funding acquisition, Formal analysis, Conceptualization. **Andrey Elchaninov:** Writing – review & editing, Supervision, Resources, Methodology, Conceptualization. **Elena Gantsova:** Writing – review & editing, Writing – original draft, Investigation, Conceptualization. **Gennady Sukhikh:** Writing – review & editing, Conceptualization. **Timur Fatkhudinov:** Writing – review & editing, Resources, Project administration, Funding acquisition, Conceptualization.

## Declaration of competing interest

The authors declare that they have no known competing financial interests or personal relationships that could have appeared to influence the work reported in this paper.

## Appendix A Supplementary data

Supplementary data.docx. The file contains images of foci obtained as a result of *in vivo* live imaging and at autopsy of the animal, images of images of full-size membranes and, also, H&E staining of liver, spleen and uterus into three groups of mice.
