# Supplementary material for: M1 macrophages as promising agents for cell therapy of endometriosis
Source: Heliyon. 2024 Aug 14;10(16):e36340. doi: 10.1016/j.heliyon.2024.e36340 (PMC11381802; doi:10.1016/j.heliyon.2024.e36340)
Supplement: Multimedia component 1 [file mmc1.docx]

- 1. **In vivo live imaging of Cy7.5-labeled endometriosis foci formed in mice 3 weeks after transplantation: abdominal view – A, lateral view – B**
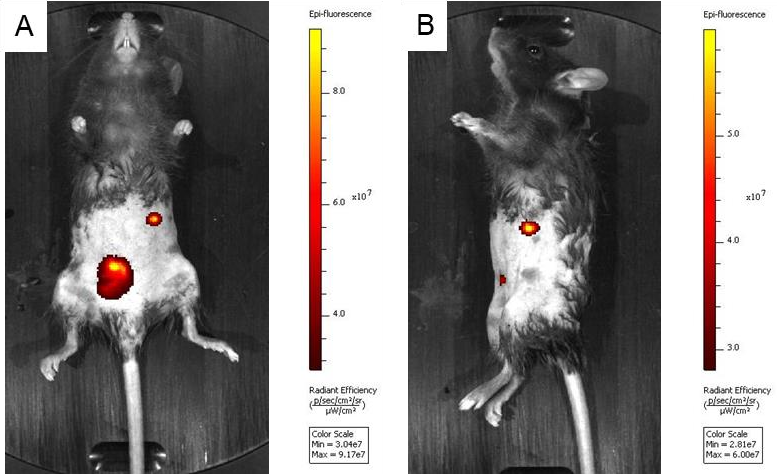

  2. **Image of endometriosis foci formed in an allogeneic model**
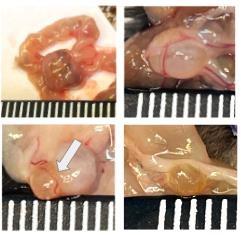

  3. **Supplementary material of Western blot**

**Fig. 1 M**

**Arginase 1**
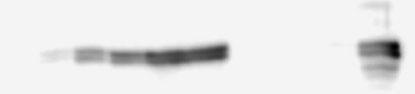


# CD86


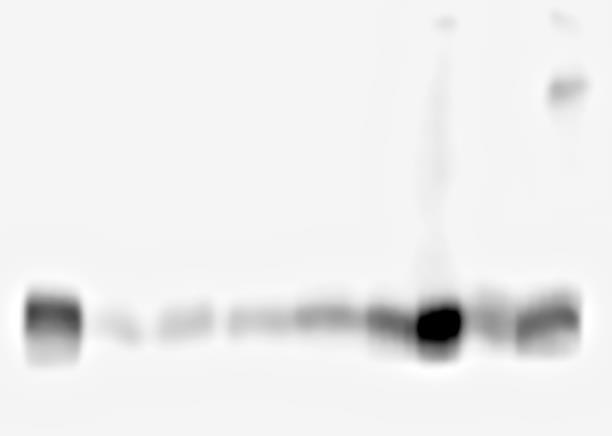


**GAPDH (Loading control)**
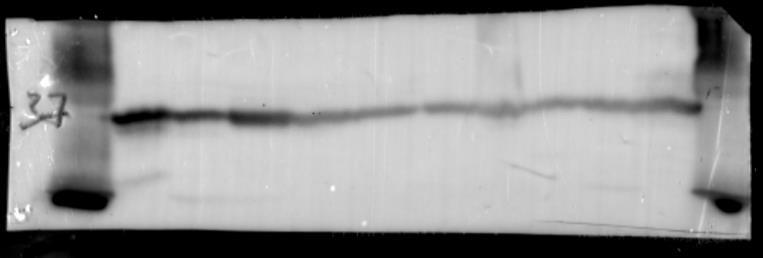


**Fig.2 B**

**GAPDH (Loading control)**
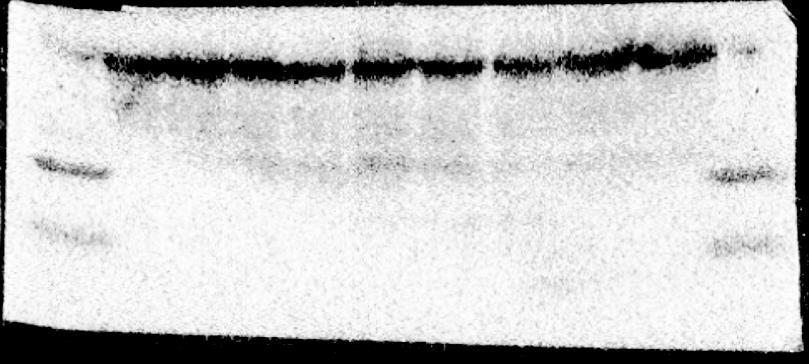


# MARCO


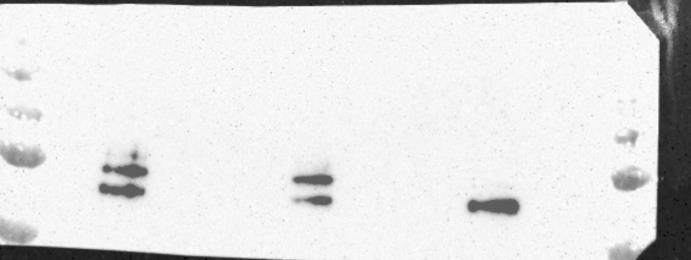


**Fig. 3 G**

**Arginase 1**
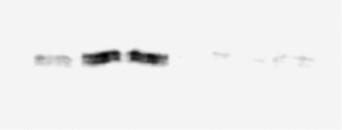


# CD86


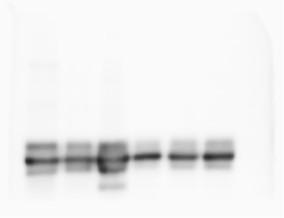


**GAPDH (Loading control)**
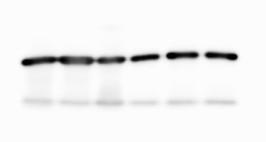


**SOCS3**
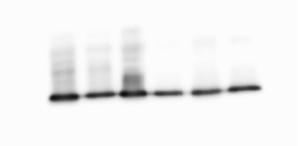


**CD206**
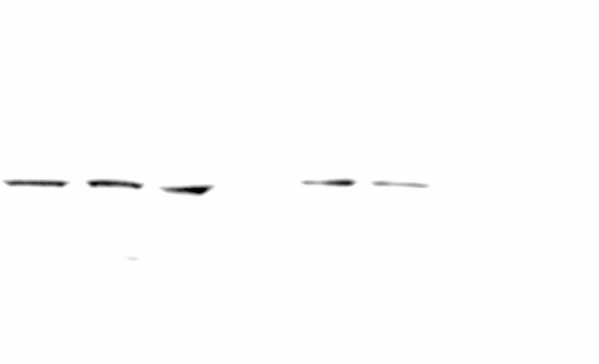


- 1. **H&E staining of liver, spleen and uterus into three groups of mice: healthy mice, mice with endometriosis and mice after therapy by M1-polarized macrophages.**

**White arrows indicate lymphatic follicles in the liver.**

**
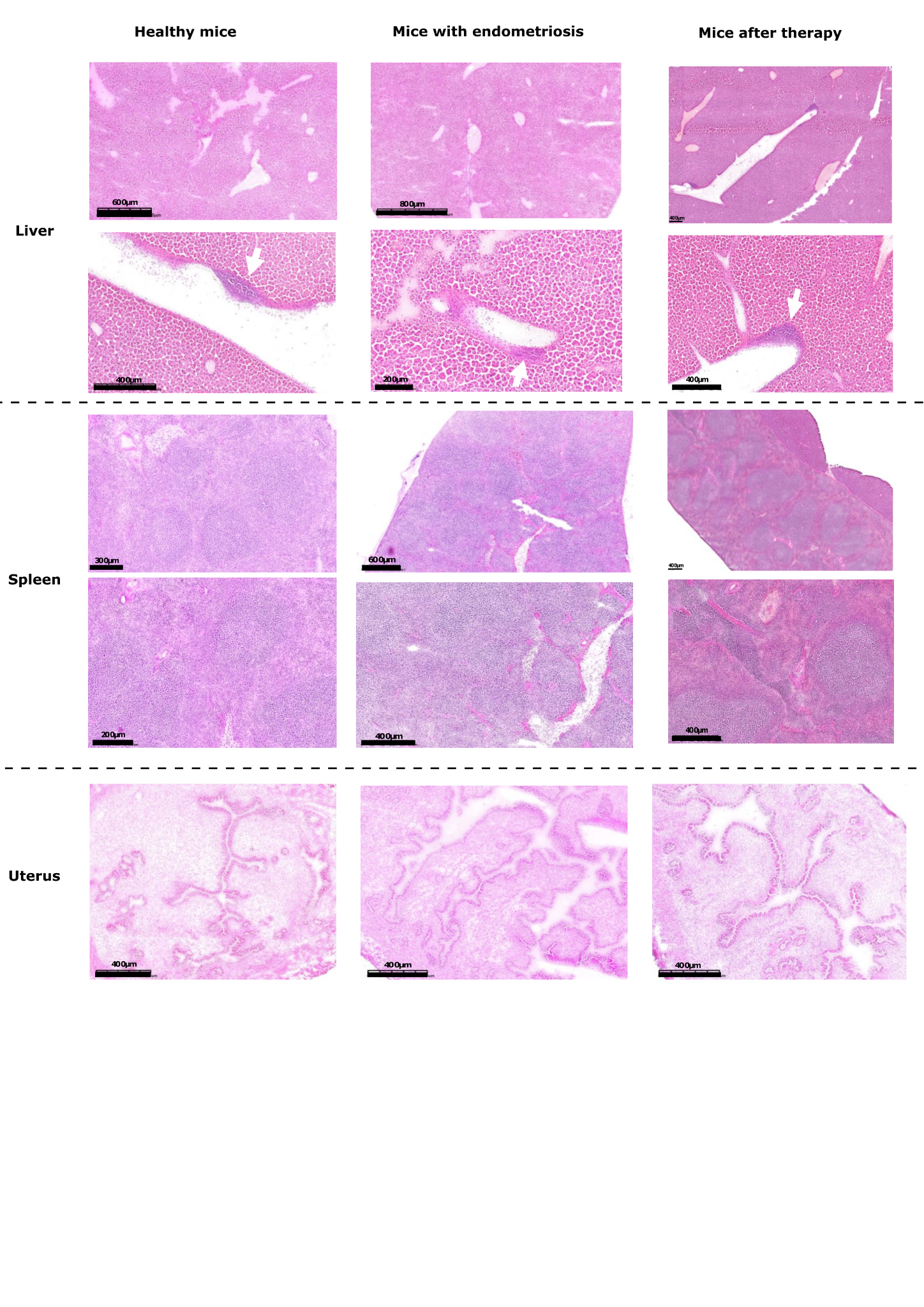
**
